# Supplementary figures and images for: Leveraging genetic interactions for adverse drug-drug interaction prediction
Source: PLoS Comput Biol. 2019 May 24;15(5):e1007068. doi: 10.1371/journal.pcbi.1007068 (PMC6553795; doi:10.1371/journal.pcbi.1007068)

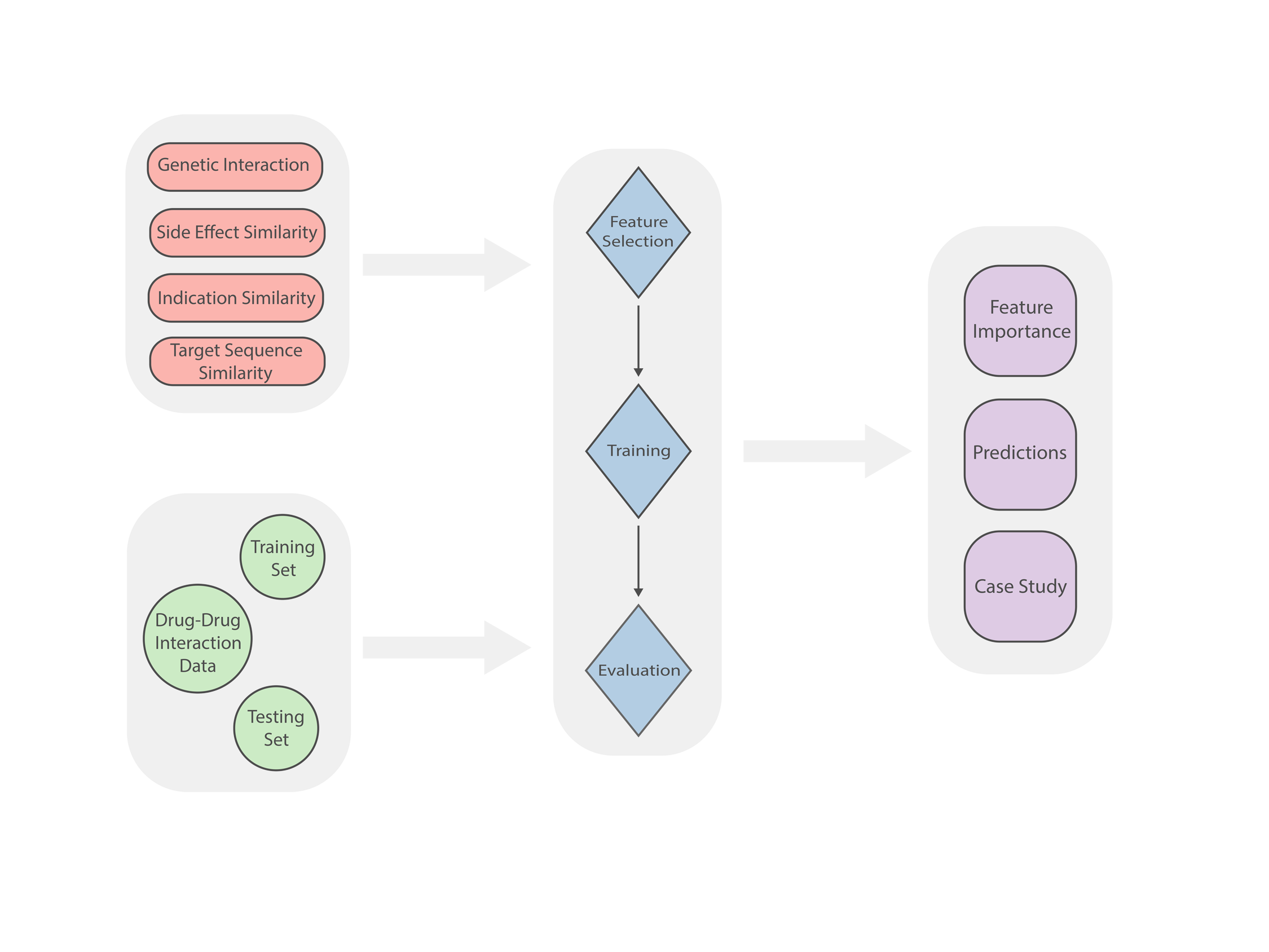

Supplement: S1 Fig — Four groups of features were calculated for each drug pair. Drug pairs were then divided into a training set and a test set. A gradient boosting-based model was built on the training set after feature selection. Model performance was evaluated on the training set using hold-out validation and also on the test set. We demonstrate the importance of our novel feature with a case study and provide novel DDI predictions at the end. (TIF) [file pcbi.1007068.s001.tif]

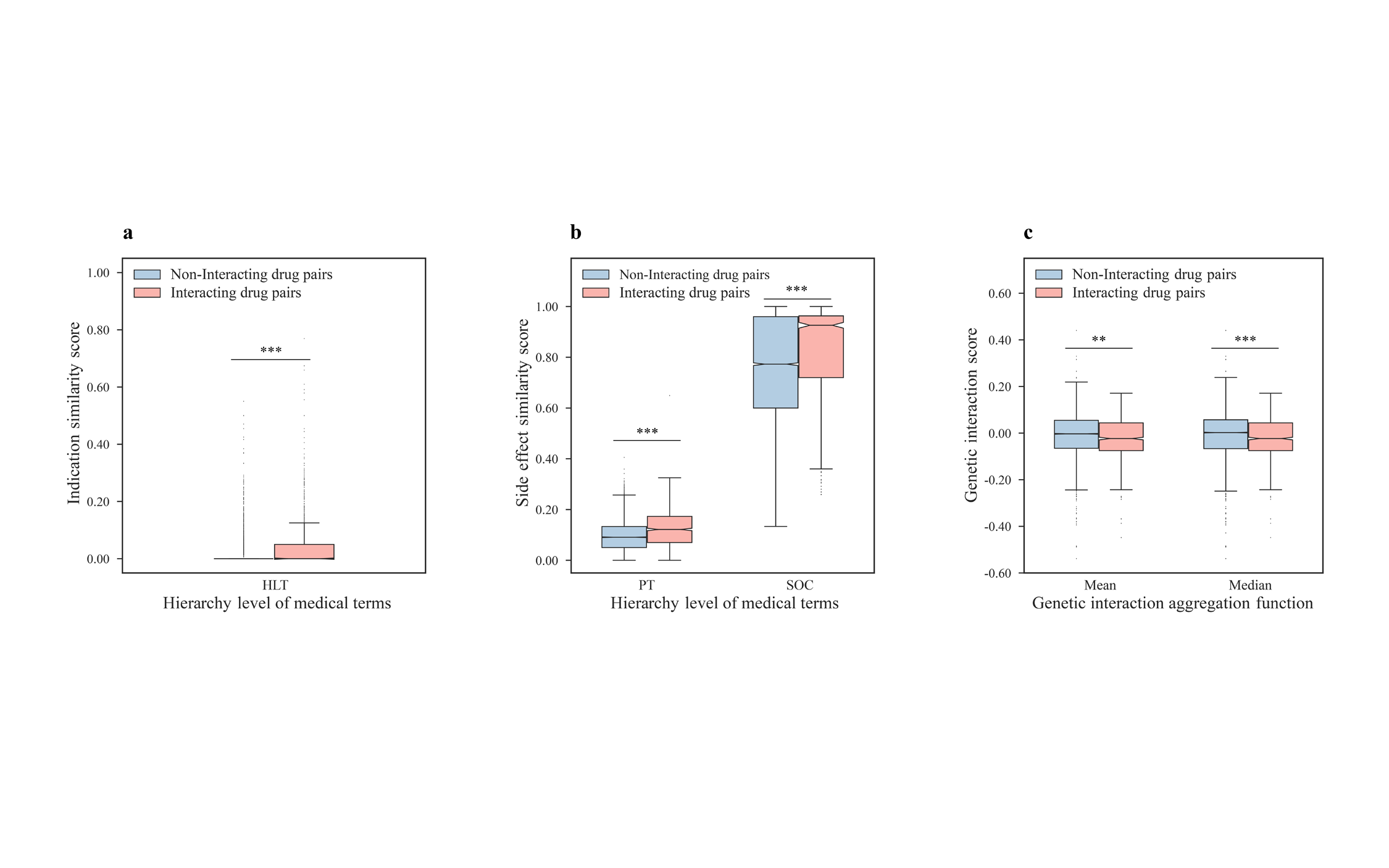

Supplement: S2 Fig — (a) Indication similarity score of hierarchy level HLT between two drugs. (b) Side effect similarity score of hierarchy levels PT and SOC between two drugs. (c) Mean and median genetic interaction score between targets of two drugs. Statistical significance was determined by the two-sided permutation test on the sample mean. * p < 0.001; ** p < 0.0001; *** p < 0.00001. (TIF) [file pcbi.1007068.s002.tif]

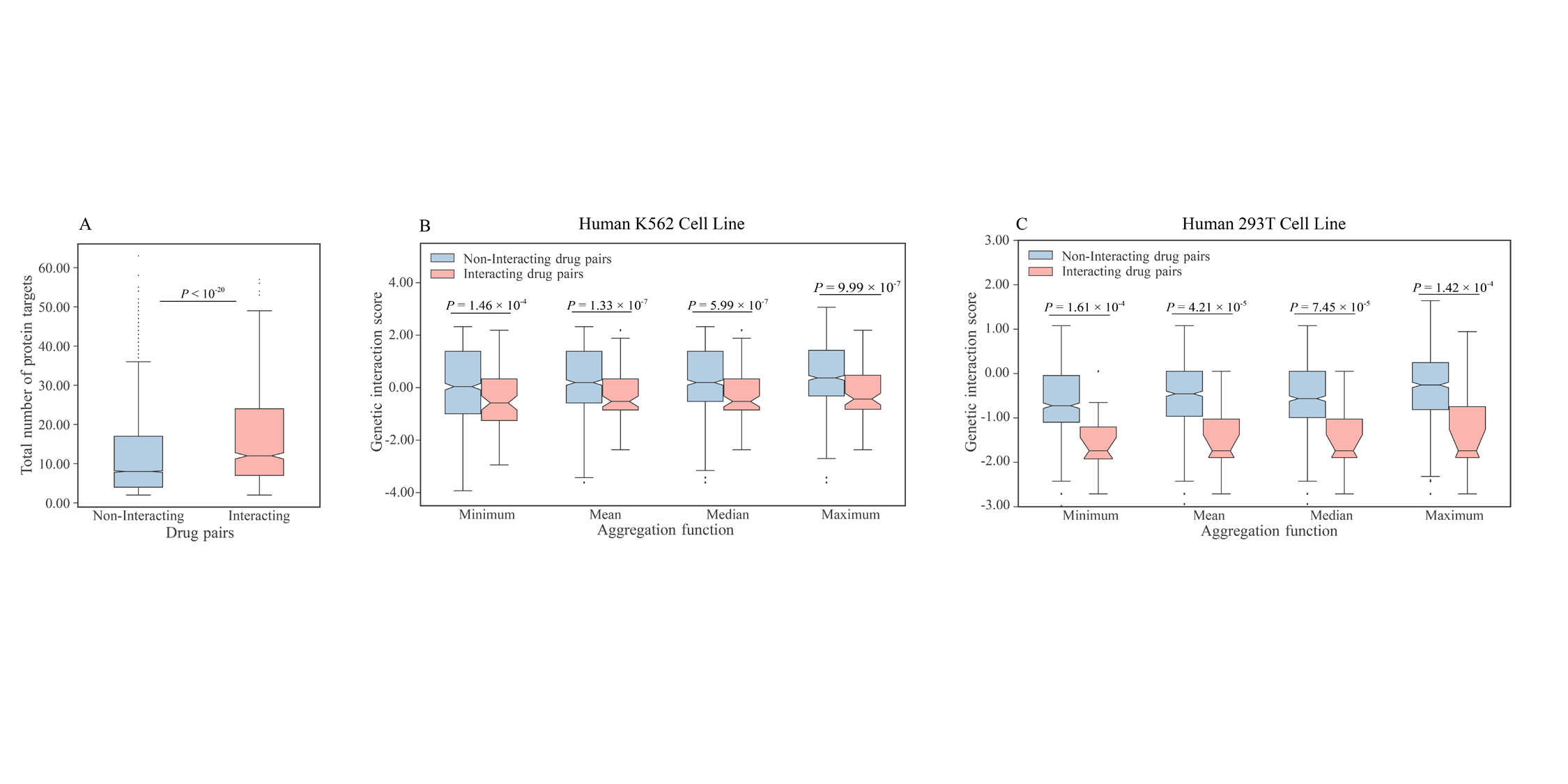

Supplement: S3 Fig — (a) The total number of protein targets between two drugs. (b) Minimum, mean, median and maximum human K562 cell line genetic interaction score between targets of two drugs. (Statistical significance determined by two-sided Mann-Whitney U test) (c) Minimum, mean, median and maximum human HEK293T cell line genetic interaction score between targets of two drugs. (Statistical significance determined by two-sided Mann-Whitney U test). (TIF) [file pcbi.1007068.s003.tif]

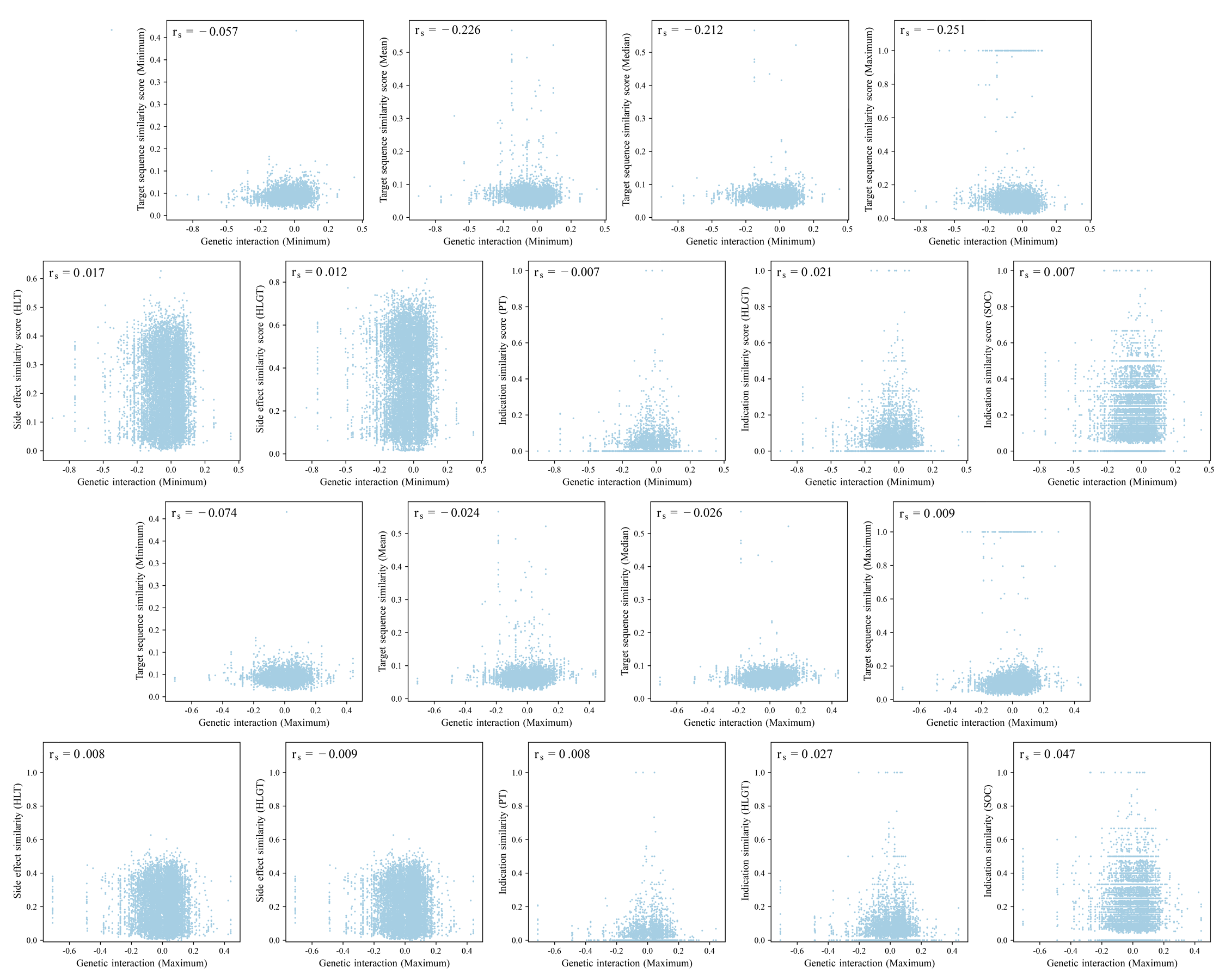

Supplement: S4 Fig — (TIF) [file pcbi.1007068.s004.tif]

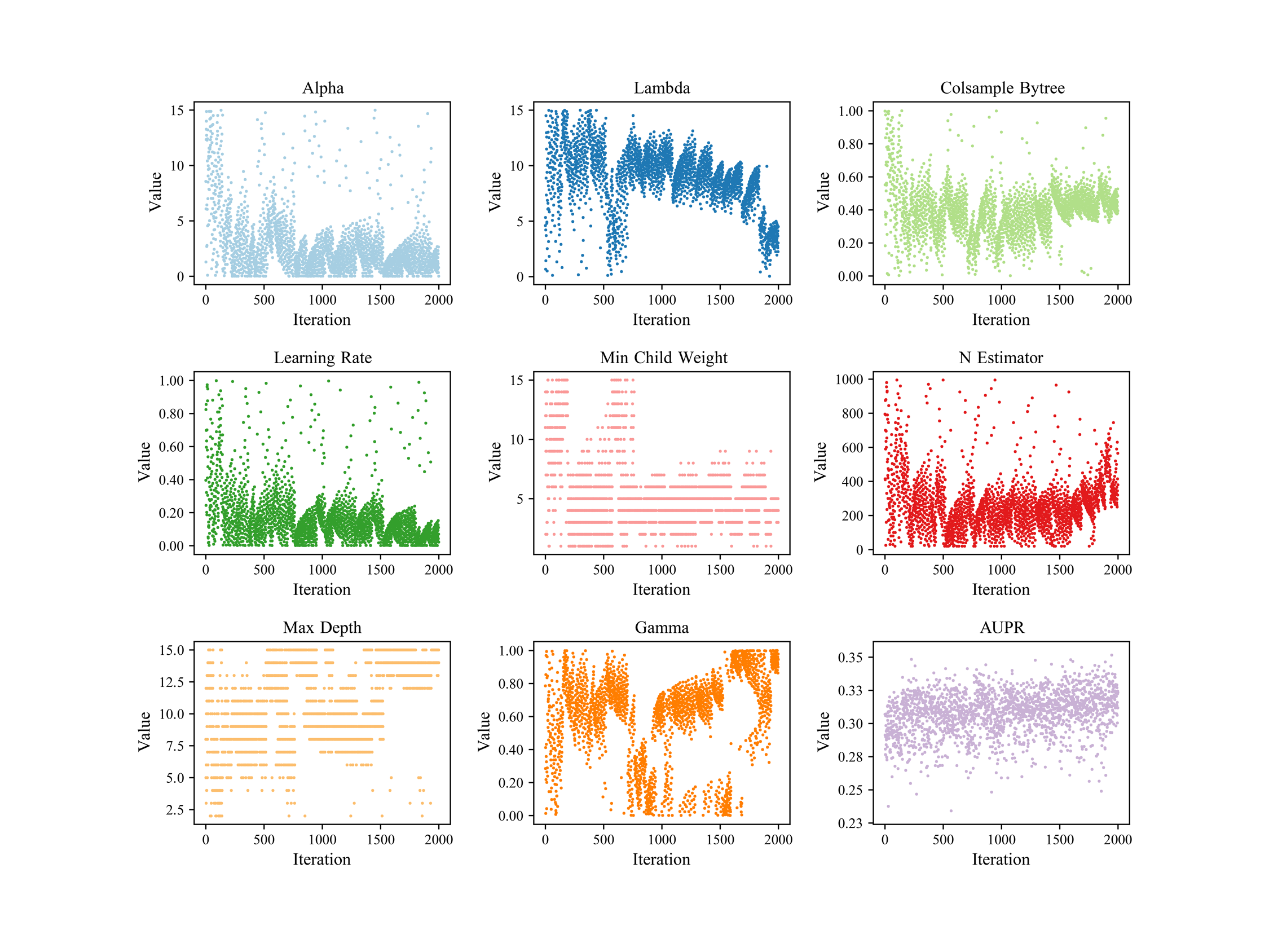

Supplement: S5 Fig — (TIF) [file pcbi.1007068.s005.tif]

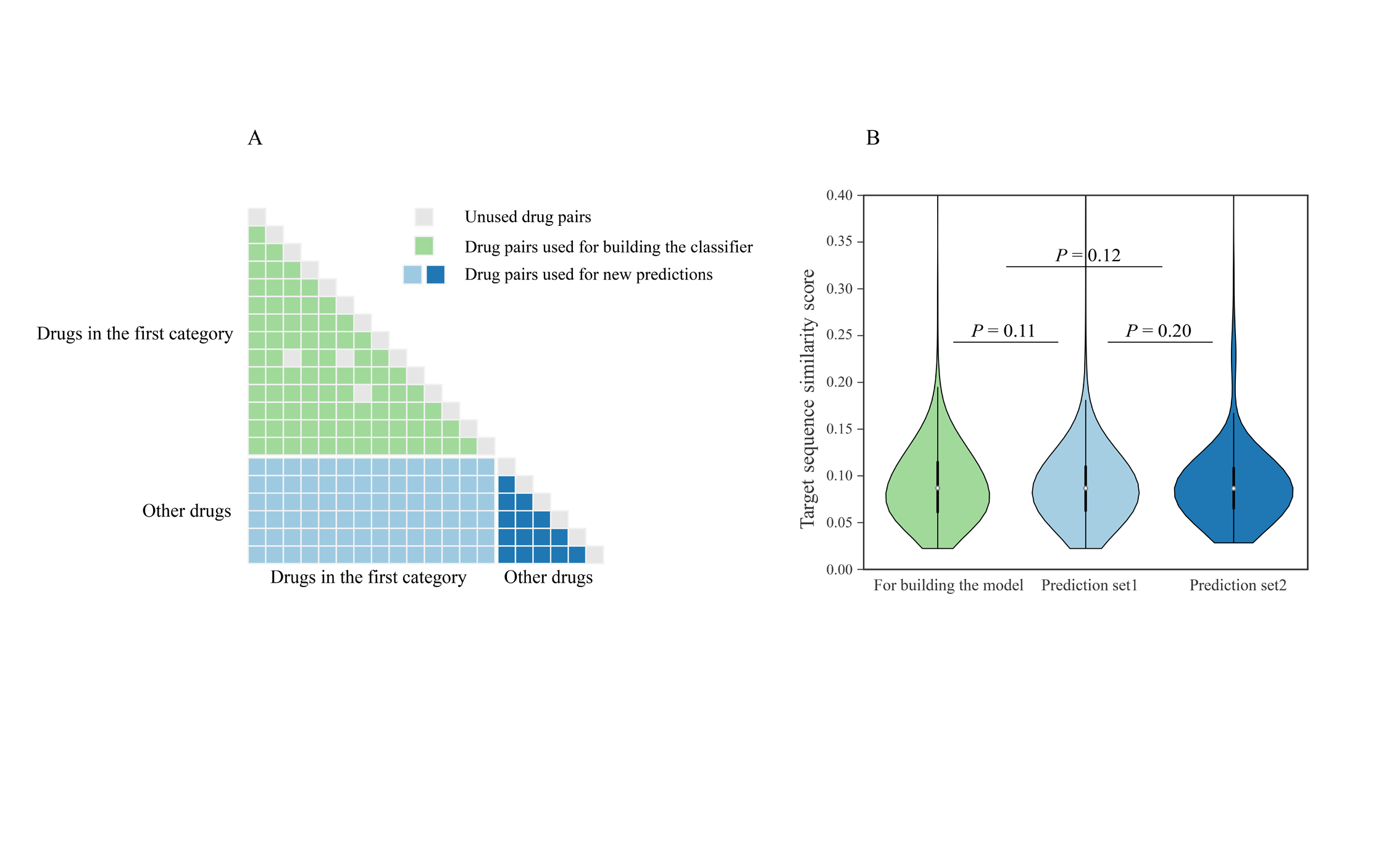

Supplement: S6 Fig — (a) All combinations between drugs that appear in the first category in DrugBank and other drugs, as well as all pairwise combinations of drugs not in the first category, are taken for new predictions. Green squares represent drug pairs used for building the classifier. Grey squares represent unused drug pairs. Blue squares represent drug pairs used for new predictions. (b) Maximum target similarity feature distribution of drug pairs used for model building (green triangular section in (a)), drug pairs where one drug appears in the dataset used for model building (blue rectangular section in (a)), and drug pairs where neither drug appears in the dataset used or model building (blue triangular section in (a)). (TIF) [file pcbi.1007068.s006.tif]
