# Supplementary material for: Leveraging genetic interactions for adverse drug-drug interaction prediction
Source: PLoS Comput Biol. 2019 May 24;15(5):e1007068. doi: 10.1371/journal.pcbi.1007068 (PMC6553795; doi:10.1371/journal.pcbi.1007068)
Supplement: S1 Table — (DOCX) [file pcbi.1007068.s007.docx]

| Category | Amount |
| --- | --- |
| The risk or severity of adverse effects can be increased when Drug A is combined with Drug B. | 117045 |
| Drug A may decrease/increase the (...) activities of Drug B. | 100440 |
| The serum concentration of Drug A can be increased/decreased when it is combined with Drug B. | 73478 |
| The metabolism of Drug A can be increased/decreased when combined with Drug B. | 41999 |
| The therapeutic efficacy of Drug A can be decreased when used in combination with Drug B. | 22358 |
